# Gradient Permittivity Meta-Structure model for Wide-field Super-resolution imaging with a sub-45 nm resolution

**DOI:** 10.1038/srep23460

**Published:** 2016-03-21

**Authors:** Shun Cao, Taisheng Wang, Wenbin Xu, Hua Liu, Hongxin Zhang, Bingliang Hu, Weixing Yu

**Affiliations:** 1 State Key Laboratory of Applied Optics, Changchun Institute of Optics, Fine Mechanics & Physics, Chinese Academy of Sciences, No.3888, Dongnanhu Road, Changchun, Jilin, P. R. China; 2 University of the Chinese Academy of Sciences, Beijing, 10039, P. R. China; 3 Key Laboratory of Spectral Imaging Technology, Xi’an Institute of Optics and Precision Mechanics, Chinese Academy of Sciences, No.17, Xinxi Road, Xian 710119, P. R. China

## Abstract

A gradient permittivity meta-structure (GPMS) model and its application in super-resolution imaging were proposed and discussed in this work. The proposed GPMS consists of alternate metallic and dielectric films with a gradient permittivity which can support surface plasmons (SPs) standing wave interference patterns with a super resolution. By employing the rigorous numerical FDTD simulation method, the GPMS was carefully simulated to find that the period of the SPs interference pattern is only 84 nm for a 532 nm incident light. Furthermore, the potential application of the GPMS for wide-field super-resolution imaging was also discussed and the simulation results show that an imaging resolution of sub−45 nm can be achieved based on the plasmonic structure illumination microscopic method, which means a 5.3-fold improvement on resolution has been achieved in comparison with conventional epifluorescence microscopy. Moreover, besides the super-resolution imaging application, the proposed GPMS model can also be applied for nanolithography and other areas where super resolution patterns are needed.

Surface plasmons (SPs) are surface electromagnetic waves trapped at the metal-dielectric interface due to the collective oscillations of free electrons of the metal^1^. Their intriguing properties such as strong localization and large in-plane momentum nature have been exploited for applications in biosensors^2^,^3^, nonlinear optics^4^, and super-resolution imaging^5^,^6^. The wave vector *k*_*sp*_of SPs can be higher than that of excited light in air (*k*_*0*_) by carefully selecting the permittivity of dielectric and metallic materials. Therefore, SPs are ideal candidates for resolution improvement purpose. Over the past decades, the SPs super-resolution has been widely studied and applied in perfect lens^7^, silver superlens^8^,^9^, and hyperlens^10^. These devices have the great potential for near-field super-resolution microscopy. In addition, SPs can also be employed in far-field microscopic imaging mode, such as standing wave surface plasmon resonance fluorescence microscopy (SW-SPRF)^11^,^12^,^13^ and structured illumination microscopy (SIM)^14^,^15^. In these two methods, standing SPs wave patterns are used as the illumination patterns. A resolution enhancement with more than twice in lateral resolution normally can be achieved in comparison with the conventional epifluorescence microscopy. Since the resolution improvement of these microscopes relies on the spatial frequency of the illumination patterns, therefore the increase in *k*_*sp*_ is critical. Previous studies show that SPs with a high frequency can be excited at the surface of a single metal layer^14^,^16^. A lot of efforts are also made to further improve the *k*_*sp*_by employing multi-layers in recent years. G. Bartal *et al*. built a 2D silver-silicon nitride-air platform to realize a 70 nm focal spot of SPs with a short wavelength^17^. However, the dielectric layer employed in this design has a rather high permittivity, which may block the near field interaction between the biological specimen and metal film. Hyperbolic metamaterials, a kind of anisotropic material in terms of permittivity that can support a very high *k*_*sp*_, are attracting more and more interest^18^,^19^,^20^,^21^,^22^,^23^ very recently. Although the hyperbolic materials can be carefully designed to obtain high wavevector of SPs, they require the challenging and costly nanofabrication processes and the error of each pair of the metal/dielectric materials would influence the accuracy of results. Therefore, less pairs or alternative forms of hyperbolic metamaterials are preferred to achieve short wavelength of SPs and to ease the nanofabrication process at the same time.

In this paper, a rather simple and elegant model, so called gradient permittivity metamaterial structure (GPMS), is presented to realize short-wavelength SPs. This kind of structure consists of three successive dielectric films with decreased gradient permittivity with thin silver films in between dielectric films. By employing finite-difference time domain (FDTD) method, the proposed GPMS is carefully simulated and analyzed to find that the standing SPs wave with an 84 nm period in one dimension can be obtained for a 532 nm incident wavelength. In addition, the possibility to improve the imaging resolution by employing the GPMS in the plasmonic structure illumination microscopic method is demonstrated theoretically. It is found that a resolution of 41 nm can be achieved in one dimension in GPMS. Finally, the physical mechanism to support short wavelength SPs in GPMS is also discussed.

## Results and Discussion

### Structure description and simulation setup

**Figure 1:**
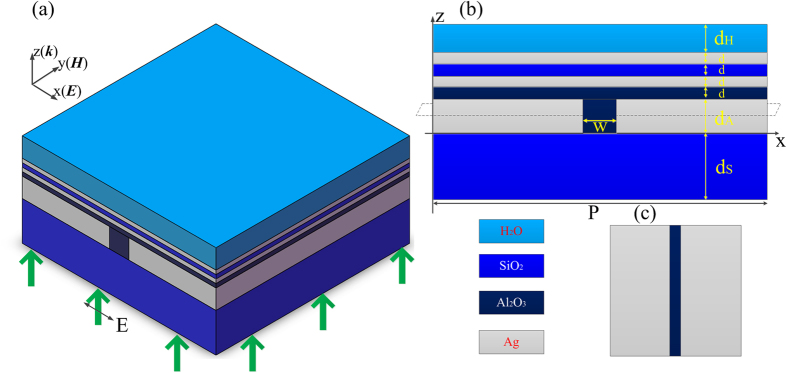
The schematic diagram of the gradient permittivity metamaterial structure. (**a**) The perspective view and (**b**) the cross sectional view of the GPMS, (**c**) The top view of the GPMS in 100 nm Ag film, marked as dashed rectangles in (**b**).

Figure 1 shows the schematic diagram of the proposed GPMS. Figure 1(a) shows the perspective view of the GPMS and the cross sectional view is shown in Fig. 1(b). As can be seen, SiO_2_ with a relative permittivity of *ε*_*S*_ = 2.13 is used as a substrate material. The thickness of the substrate is set to be *d*_*S*_ = 200 nm. Next, a silver layer (the permittivity is −11.75 + 0.37i^24^ at incident wavelength of 532 nm) with a thickness of *d*_*A*_ = 100 nm was deposited on the top of the substrate. In order to generate surface plasmon waves, a subwavelength slit array with a width of *W* = 100 nm for each slit is perforated in silver layer and the slit is then filled with Al_2_O_3_ material (*ε*_*Al*_ = 3.138). The slit array has a period of *P* = 1 μm. It is because the Fourier transform of the periodic slit array includes broadband wave vectors so that the incident light can be coupled into SPs wave when the momentum matching condition is met. The feature of the layer (marked as a dashed rectangle in Fig. 1(b)) is shown in Fig. 1(c). The reason why the period of the slit array is chosen as 1 μm is because the propagation length of the surface plasmon polariton is calculated to be 1 μm by using the Equations (2.6) and (2.11) in reference 1. This will make sure the whole working field can be covered by the standing wave of SPs. On the top of the silver slit array, GPMS with four successive layers of Al_2_O_3_, Ag, SiO_2_ and Ag with the same thickness of *d* = 20 nm were coated one by one. In accord with the aqueous environment of many biological samples, a 100 nm water film was introduced on the top of the structure, which serves as the objective plane in the structure illumination microscopy.

The finite-difference time domain (FDTD) simulation software (Lumerical FDTD Solutions) was used to model and analyze the GPMS. Three-dimensional simulations were performed with a TM polarization plane wave with a wavelength of *λ*_*0*_ = 532 nm incident in *z* direction. The inset of Fig. 1(a) shows the direction of incident visible EM waves with respect of the cross section of the designed GPMS.

**Figure 2:**
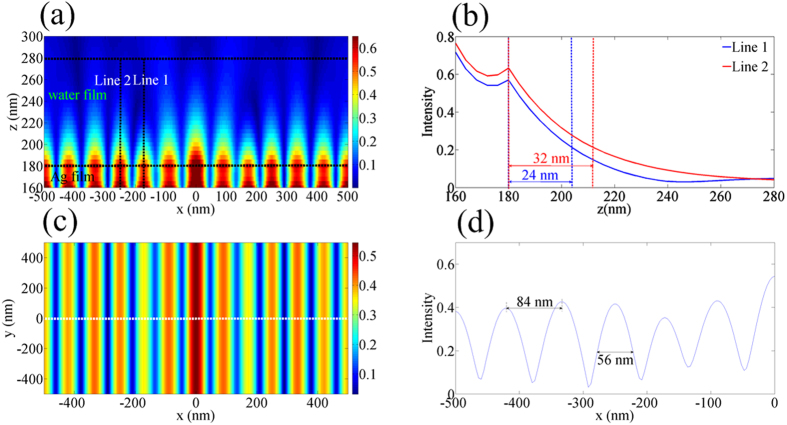
The distribution of the standing SPs in the water film of the GPMS. (**a**) The distribution of *x*-component of the electric field in *y* = 0 plane. (**b**) The intensity of the profile of electric field along the lines in (**a**) blue curve stands for Line 1 and red curve represents Line 2. (**c**) The distribution of *x*-component of the electric field in *z* = 192 nm plane. The color bar in (**a**) and (**c**) means the intensity of the electric field. (**d**) The cross section of electrical field intensity distribution at the white dashed line in (**c**).

Frequency-domain field and power monitors in FDTD were used to investigate the distributions of the electric field at different planes and the results are shown in Fig. 2. Figure 2(a) shows the *x*-component of the electric field at plane *y* = 0, i.e. the interface of the uppermost silver layer and the water film. The color bar represents the electric intensity. It can be seen clearly that the standing wave of the surface plasmon wave is generated in the Ag film and the water due to the interference of the SPs excited by the subwavelength metallic slit array. However, the intensity distribution of the SP standing wave is not very uniform in the water layer. After that, the electric intensity along two lines (Line 1 and Line 2 are black dashed lines in Fig. 2(a)) was extracted and is shown in Fig. 2(b). As can be seen, the intensity for Line 2 is larger than that for Line 1 in one unit cell of GPMS. In addition, the intensity of SPs decreases with the form of exponential attenuation when SPs wave propagates from *z* = 160 nm (the interface of the SiO_2_ and Ag film) to *z* = 180 nm (the interface of the Ag and water layer). At *z* = 180 nm, the SPs wave has a sub-peak, which means the electric field has been enhanced a little bit. The penetration depth of SPs wave inside the water film can also be obtained. From the blue curve in Fig. 2(b), the standing wave in Line 1 has a penetration depth of about 24 nm. While for Line 2, the penetration depth is about 32 nm, which is a little bit larger than that of Line 1. In general, the penetration depth of SPs wave is in the scale of about 30 nm. The distribution of the *x*-component of electricity at plane *z* = 192 nm (12 nm above the interface of the Ag/water film) is shown in Fig. 2(c). Figure 2(d) is the cross section of the intensity distribution along the white dashed line in Fig. 2(c). Please note that only half of the line is plotted due to the symmetry of the structure. From this picture, the period of the standing wave of SPs is determined to be 84 nm and the full width at half maximum (FWHM) is about 56 nm. Consequently, the period of the SPs interference pattern can be obtained is only 0.16 *λ*_*0*_ for our GPMS model.

### GPMS for super-resolution Imaging

**Figure 3:**
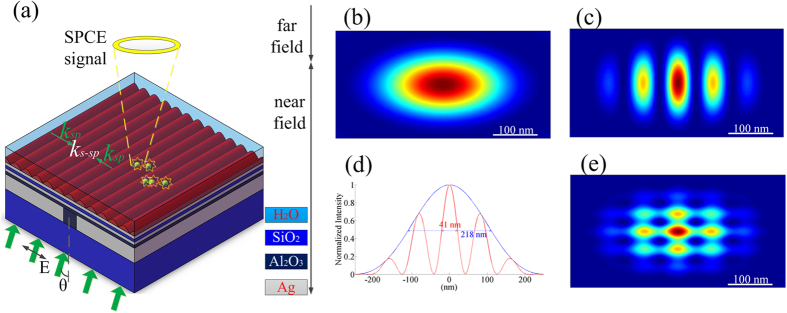
The schematic diagram of the GPMS used in SIM and the simulation results of its imaging performance. (**a**) Optical configuration of SPs generated by GPMS. The standing interference pattern, which is generated by two adjacent counter propagating SPs, is used to excite the quantum dots (or fluorescent beads) in the water film and point spread function of (**b**) a diffraction-limited system, (**c**) *x*-direction reconstructed image, (**d**) FWHM comparison between conventional epi-fluorescence microscope image (blue curve) and the super-resolution image using the GPMS (red line), (**e**) both *x* and *y* direction reconstructed image.

The proposed GPMS could be applied for super-resolution imaging with a plasmonic structure illumination microscopic mode. The schematic diagram of the process of the GPMS used in super-resolution imaging is shown in Fig. 3(a). In order to demonstrate its capability in resolution improvement, the image of a quantum dot (QD) was calculated. The QD object was deposited on the interface of the top Ag and water films. The standing SPs pattern serves as the structured illumination pattern to illuminate the QD object and the surface plasmon-coupled emission (SPCE) signal of the QD can be recorded on a charge-coupled device (CCD) camera in far field (see Fig. 3(a)). To generate the image reconstruction, at least three intermediate images with different phase of the illumination pattern need to be recorded^25^. The phase changes can be obtained through tuning the incident angle *θ* of the incident light^15^. Here, a sequence of three images with 0, 120, −120° phases were calculated. By rotating the GPMS along *z* axis, the orientation of the illumination pattern along both *x* and *y* directions can be adjusted. The numerical algorithm in standing-wave total internal reflection fluorescence (SW-TIRF) imaging was used to reconstruct the high-resolution image^25^.

In our simulation model, a 10 nm QD, with an emission wavelength of 600 nm was used to achieve the point spread function (PSF) of the standard system and characterize the resolution of the GPMS. The immersion oil objective with a NA of 1.42 was considered in the numerical model. The results are shown in Fig. 3. Figure 3(b) shows the image of the PSF of the QD with a conventional homogeneous illumination. Figure 3(c) shows the reconstructed image of the QD by using the standing SPs pattern in *x*-direction generated in GPMS as the illumination light. Figure 3(d) shows a comparison of the PSF profile of Fig. 3(b,c). From the comparison, one can find that the FWHM of the conventional epi-fluorescence microscopy is about 218 nm and the FWHM is only about 41 nm for GPMS illumination microscopy. This means that the imaging resolution of GPMS illumination microscopy is about 5.3 folds of that conventional epi-fluorescence microscopy. This result is better than SIM^26^ and previous reported PSIM^14^,^15^,^27^. It should be noted that two-dimensional enhancement in imaging resolution of the QD can also be obtained by using the SPs pattern generated in GPMS to illuminate the QD in both in both *x* and *y* directions. This can be achieved by dynamically controlling the orientation of the standing SPs wave pattern via the rotation of GPMS. Figure 3(e) shows the reconstructed image of the QD by using the standing SPs pattern to illuminate in both *x* and *y* directions. Apparently, the imaging resolution has been improved in both *x* and *y* directions in Fig. 3(e). However, as can be seen clearly, there exist sidelobe artifacts surrounding the central spots in Fig. 3(c,e), similar to other super-resolution methods based on interference method^28^,^29^. These artifacts can be easily removed by using appropriate numerical processing method so that the image quality can be improved further^30^.

### Confirmation of GPSM by analytical computation

**Figure 4:**
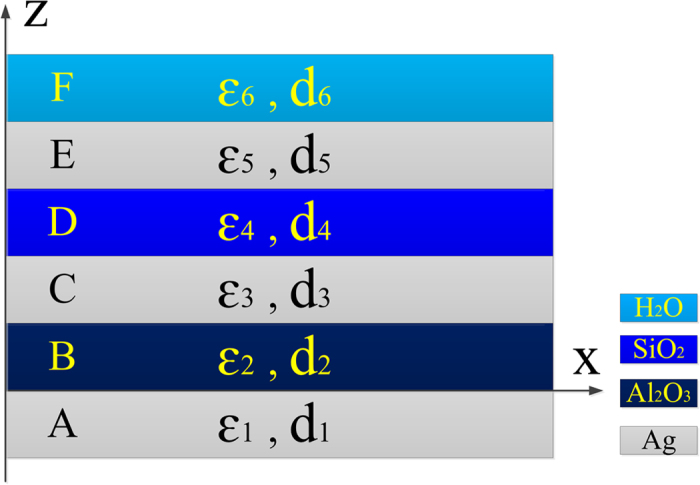
The schematic diagram of the analytical model for GPMS. Here *ε*_*i*_ and *d*_*i*_ stand for the permittivity and thickness of the material in the i*th* layer respectively.

To further understand the physical mechanism of the GPMS, the SPs wavevector supported by it is derived analytically through the Maxwell equations. To follow the GPMS, an analytical computing model is built as shown in Fig. 4 and the permittivity of the material in each layer is shown in the figure. Please note that the coordinate system of *x-z* axis is different from that of the GPMS in Fig. 1 due to the convenience in the theoretical analysis. A TM polarized electromagnetic wave incidents along *z* axis. It is assumed that the SPs wave is excited at the interface between Layer 1 and Layer 2.

From the Maxwell equations, the component of the electric and magnetic field can be acquired.

In the Region A (z < 0),


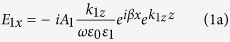



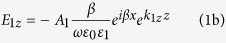



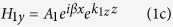


and in Region B-E,


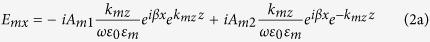



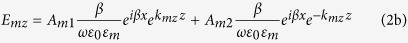



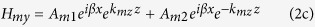


where m = 2–5, in Region F, the component of the electric and magnetic field can be derived as follows,


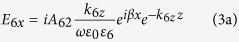



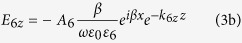



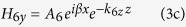


With the boundary conditions imposed at five interfaces, i.e. the *x*-component of the electric field and the *y*-component of the magnetic field must be continuous. Then at the interface of *z* = 0, *E*_*1x*_* = E*_*2x*_, *H*_*1y*_* = H*_*2y*_, one can get the following equations,


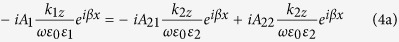



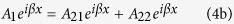


Then the following equation can be obtained,


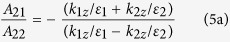


Similarly, one can get the following equation at *z* = d_2_ + d_3_ + d_4_ + d_5,_


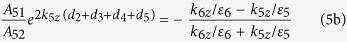


At the interface *z* = d_2_ + d_3_ + d_4_,


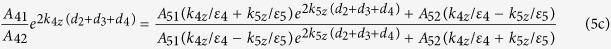


At *z* = d_2_ + d_3_,


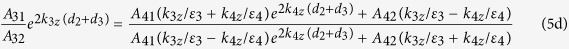


And at *z* = d_2_,


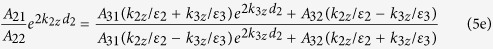


Assume *A* = *A*_*21*_/*A*_*22*_, *B* = *A*_*51*_/*A*_*52*_, *C* = *A*_*41*_/*A*_*42*_, *D* = *A*_*31*_/*A*_*32*_, then one can get the following equations,


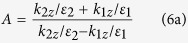



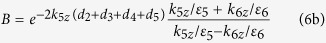



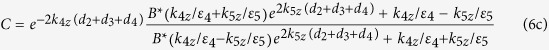



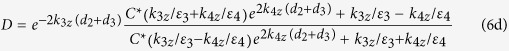



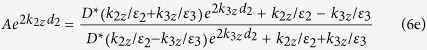


In above equations,


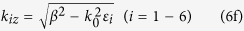


Where *k*_*iz*_ stands for the component of the wave vector of the SPs in the *i*th layer that perpendicular to the interface, and *β* is the component parallel to the interface in the GPMS. The symbol *k*_*0*_ represents the wave vector of the incident light and d_*i*_ (*i* = 1–6) stands for the thickness of the *i*th layer. Subsequently, the different permittivity of each layer in GPMS were taken into account and the wave vector of SPs in GPMS can be calculated by combing Equations (6a–6f), 18, 19, 20, 21, 22 to be *β = *0.0377 rad/nm. As a result, the wavelength of SPs in GPMS was calculated to be 166 nm. Therefore, the period of the interference standing wave of SPs is half of it, i.e. 83 nm, which is almost identical to that obtained by the rigorous numerical simulation. As a result, the validity of the GPMS model is confirmed by both numerical and analytical methods.

Moreover, the GPMS can be tuned with a great flexibility due to there are many parameters, such as the thickness of the dielectric materials and the permittivity of the metal and dielectric can be changed so that the wavelength of the SPs can be tuned. Here, Ag is chosen as the metallic material due to its small propagation loss and hence SPs with a large propagation length can be supported. In our GPMS, though the metallic silt array were used to generate interference SPs, other coupling elements such as half circle silt^17^ and the computer designed metasurface^31^ can also be used to generate additional form of SPs interference pattern. By employing GPMS, super-resolution imaging in a very large wide field can be realized due to the periodic property of the slit array. From the color bar in Fig. 2(a), the intensity of the standing SPs in the water film is about 0.6 *I*_*0*_ (*I*_*0*_ is the intensity of incident light), which is much stronger than that obtained by traditional multilayer metamaterials^18^. This benefits the application in the imaging of the biological samples by eliminating the use of a high power laser source. However, the penetration depth of the standing SPs in the water medium is only about 30 nm, which is much shorter than that in the ordinary Ag-air structure.

## Conclusions

In summary, a gradient permittivity metamaterial structure (GPMS) was demonstrated in this work. The GPMS features a gradient permittivity by employing only a few layers of alternative dielectric/metallic films. In comparison with traditional multilayer metamaterial structure, GPMS is more simple and elegant but still can support SPs wave with an even higher wavevector. The validity of the GPMS on the support of short wavelength SPs wave was proven by employing both rigorous numerical and analytical methods and the results show a good agreement for both methods. It is found that the period of the SPs interference pattern is only about 0.16 of the wavelength of the incident light. The potential application of this deep subwavelength SPs interference pattern on super-resolution imaging was also discussed. It is shown that the reconstructed quantum dot image shows a 5.6-fold improvement on the resolution in comparison with that of the conventional epifluorescence microscopy. In addition, the wavelength of the standing SPs can be tuned via changing the parameters of the GPMS. All of these advantages of GPMS promise great potential applications in the field of super-resolution biomedical imaging as well as in nanolithography.

## Methods

All the numerical simulations of the GPMS in this paper were done with commercial Finite Difference Time Domain (FDTD) software (FDTD Solutions) developed by Lumerical Solutions, Inc. The high accuracy mesh type, i.e. the 6th level of the auto non-uniform mesh type, was used to ensure a reliable result. A FDTD simulation region of 1 × 1 × 1 *μm*^3^ with Periodical Boundary (PB) in both *x* and *y* directions and Perfectly Matched Layers (PML) as absorbing boundary conditions in *z* direction was used to calculate and analyze the GPMS. Moreover, a finer mesh region with small cubes of 4 × 4 × 4 *nm*^3^ was applied in the region where SPs exist while a coarse meshing was applied elsewhere. Frequency-domain field and power monitors in FDTD at *y* = 0 nm, *z* = 192 nm were used to investigate the distributions of the electric field at these planes.

## Additional Information

**How to cite this article**: Cao, S. *et al*. Gradient Permittivity Meta-Structure model for Wide-field Super-resolution imaging with a sub-45 nm resolution. *Sci. Rep.* **6**, 23460; doi: 10.1038/srep23460 (2016).
